# An Algorithm Predicting the Optimal Mechanical Response of Electronic Energy Difference

**DOI:** 10.1021/acs.jctc.3c00490

**Published:** 2023-09-05

**Authors:** Alejandro Jodra, Cristina García-Iriepa, Luis Manuel Frutos

**Affiliations:** † Departamento de Química Analítica, Química Física e Ingeniería Química, y Grupo de Reactividad y Estructura Molecular (RESMOL), Universidad de Alcalá, Alcalá de Henares, 28806 Madrid, Spain; ‡ Instituto de Investigación Química ‘‘Andrés M. del Río’’ (IQAR), Universidad de Alcalá, Alcalá de Henares, 28806 Madrid, Spain

## Abstract

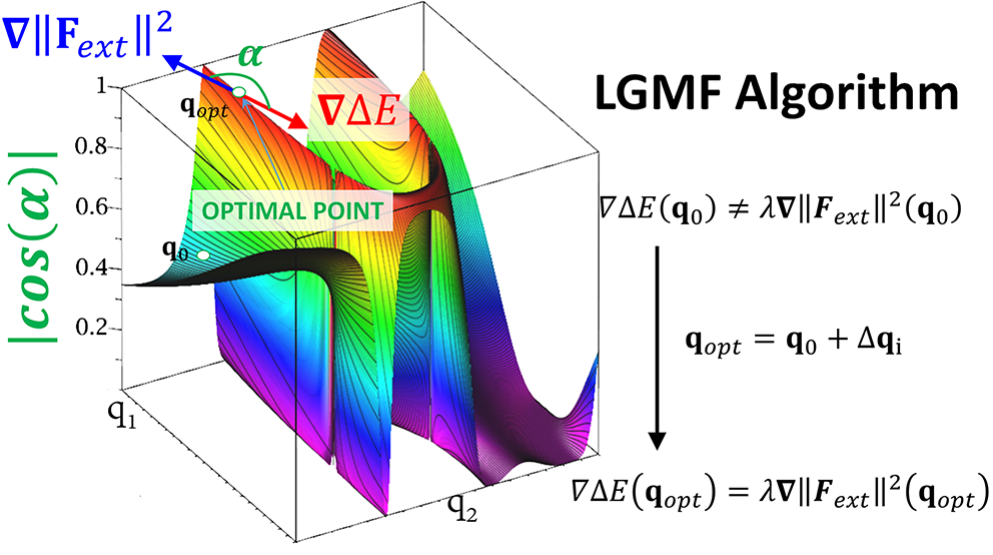

The use of mechanical forces at the molecular level has been shown to be an interesting tool for modulating different chemical and physical molecular properties. The so-called covalent mechanochemistry deals with the application of precise mechanical forces that induce specific changes in the structure, stability, reactivity, and other physical properties. The use of this kind of force to modulate photophysical properties and photochemical reactivity has also been studied. Nevertheless, the general problem of mechanical modulation of the energy gap between two electronic states has been addressed only with the development of simple theoretical models. Here, we develop and implement an algorithm providing the *Largest energy Gap variation with Minimal mechanical Force* (LGMF) that allows the determination of the optimal mechanical forces tuning the electronic energy gap, as well as to identify the maximum mechanical response of a molecular system to the application of any mechanical stimulus. The algorithm has been implemented for diverse molecular systems showing different degrees of flexibility. The phyton code of the algorithm is available in a public repository.

## 1 Introduction

The use of mechanical forces to induce chemical reactions and control molecular properties is an emerging field known as mechanochemistry.^1^ These forces develop mechanical work on the molecular systems, eventually affecting their chemical and physical properties. Unlike traditional control and activation methods such as thermochemistry, photochemistry, or electrochemistry, mechanochemistry offers a unique and efficient way to control reactivity and molecular properties due to its vectorial nature. Mechanochemistry has been used in industry for many years, such as in ball milling, compression, or mastication of polymers and solids.^2^ With the development of methods for manipulating single molecules, it has become possible to study the role of external mechanical forces at the molecular level.^3^ The application of specific forces applied in a very controlled way, the so-called covalent mechanochemistry, offers the opportunity to finely tune molecular properties, including chemical reactivity.^4^

There are different experimental techniques for implementing controlled forces at the molecular level. Some examples are AFM-derived techniques,^5^ sonication,^6^ or force probes/molecular switches.^7^ The particular application of external mechanical forces to biological systems has led to the development of mechanobiology.^8^ Additionally, the implementation of mechanical forces in materials led to the development of mechanoresponsive materials.^9^

Covalent mechanochemistry has also been theoretically studied, and different models and methods have been developed, such as “constrained geometries simulate external force” (CoGEF),^10^ “force-modified potential energy surface” (FMPES),^11^ “external force explicitly included” EFEI^12^ and “enforced geometry optimization” EGO.^13^ These methods have been implemented for the computational study of many different mechanochemical problems.

The control of the light absorption and emission wavelengths in molecular systems is a central issue in photophysics. It is very relevant in many different contexts like in the control of wavelength emission in organic light-emitting diodes (OLEDs),^14^ the control of absorption spectra in *E*/*Z* photochromic switches to improve their photoconversion yield,^15^ or in the case of photodynamic therapy, where the photosensitizers have to exhibit absorption in the near-infrared to efficiently penetrate in tissues.^16^

In order to modulate the absorption/emission properties of molecular systems, or more general, the energy difference between two electronic states, strategies have been employed, most of them relying in the molecular design.^15^ In this context, previous results show that mechanical control of the energy difference between electronic states is also possible by applying specific force pairs in the azobenzene photoswitch.^17^ Additionally, the effect of the mechanical forces has been shown to also affect the photoreactivity, making possible the mechanical control of the photoproduct outcome,^18^ or the modulation of photoisomerization and fluorescence quantum yields in molecular photoswitches.^7,19^ Energetic parameters, like the fraction of photon energy converted to chemical energy in molecular solar thermal systems, can also be tuned with mechanical control.^20^ All these mechanical effects in processes involving excited states rely on changes of the potential energy surface (PES) topology induced mechanically, making it also possible to modulate the conical intersection topography.^21^

Additionally, mechanochromic materials based on the use of mechanophores usually inserted in polymer matrices has been designed with different applications, like inducing/controlling fluorescence,^22^ color changing,^23^ or energy transfer.^24^ The reliant nature of this mechanochemical control can be ultimately reduced to the molecular mechanophore properties.^3^

The computational study of mechanical energy gap modulation relies on the concept of optimal forces. These forces correspond to the largest variation in the energy gap when a mechanical force of a given magnitude is applied. They have a special relevance for two main reasons: first, they define the largest mechanical response of the energy gap for the system, and second, they permit identification of the relevant mechanical coordinates tuning the specific energy difference between the considered electronic states. In order to computationally predict the mechanical modulation of the energy gap in a molecular system, some analytical models have been developed in order to predict the mechanical response of the excitation energy of a chromophore,^25^ or the whole absorption spectrum including the relative positions and intensities of different bands.^26^ These methodologies are based on a second-order approach of the PES (initial and final states) around the minimum of the initial electronic state (i.e., usually the ground-state in the case of excitation energy). Using this approach, the variation of the energy gap as a function of the applied force is obtained, and imposing a minimum force magnitude (i.e., constrained optimization) yields the optimal solutions being found.

The use of a second-order approach to describe the PES has some well-known limitations related with the validity of the approximation for large structural displacements: (*i*) If the force is applied along low-frequency modes, the numerical error could be significant, even for small forces, (*ii*) the usual use of Cartesian coordinates may mix low- and high-frequency modes inducing a numerical error in the predictions, and (*iii*) in general, for relatively large forces (i.e., involving large displacements), the error could be significant since the second-order approach may be no longer valid.

Taking into account these limitations, an algorithm exploring complete PES, and producing exact results, overcomes the limitations above-mentioned in the prediction of mechanical modulation of the energy gap. Here, we describe and implement an algorithm with these characteristics based on the local approximation of PES determined for specific points (i.e., structures) in the configurational space. This algorithm provides the highest theoretical mechanical response of the energy gap and the optimal forces permitting this control. This force can be usually approached as a force pair, or a sum of different force pairs.^25^

## 2 Methods

All relative energies between surfaces have been performed within the isometric approximation using the TD-DFT level of theory for electronic structure calculations implemented in Gaussian09 suite of program.^27^ In particular, for H_2_O, the calculations were done using the B3LYP functional with the 6-31G* basis set. In the case of semibullvalene (SBV), the functional chosen was the CAM-B3LYP^28^ together with the 6-31G* basis set. For pyrene, the functional chosen was B3LYP with the 6-31+G** basis set. Previous results concerning both SBV^25^ and pyrene^26^ show the good agreement of these functionals in predicting the experimental excitation energies. All of the gradients have been computed analytically, while the Hessians of the minimum energy structures have been computed analytically for the ground state and numerically for the excited states.

## 3 Results and Discussion

Here, we address the problem of mechanical control of excitation/emission energy or, in general, the energy difference between two electronic states. We develop an algorithm exploring complete PESs that permits predictions to be made whose validity is only limited to the quality of the electronic structure method selected for the study.

The computational problem of mechanical control of excitation/emission energy tuning can be stated in simple terms: finding the optimal force that promotes a change (i.e., increase or decrease) in the energy gap. By optimal force, we will always refer to the force of minimum magnitude provoking a given variation in the energy gap.

### 3.1 Description of the LGMF (Largest energy Gap variation with Minimal mechanical Force) Algorithm

Here, we describe the LGMF algorithm providing the largest energy gap variation with minimal mechanical force (LGMF), its structure, and its implementation. First, the local approach of the potential energy surfaces is presented. Then, the inclusion of mechanical forces is discussed, after which the mathematical condition for optimal solutions for any force magnitude is analyzed, including the case of multiple solutions related to symmetry breaking. Then, the practical implementation of the algorithm is discussed in detail, showing the different steps and how the potential energy surfaces are explored. In the following, two electronic states will be considered, labeled as “0” and “1”, i.e., the initial and final states, respectively.

#### 3.1.1 Second-Order Approach of Potential Energy Surfaces

In order to computationally predict the structural response of a molecular system to a given external force, it is necessary, at least, to have a second-order approach of the PES. This is because the internal forces are the first derivatives of the potential energy with respect to coordinates, and consequently, the simplest (i.e., first order) approach of forces in terms of coordinates is achieved with the said second-order approach of the PES. Therefore, in order to implement any algorithm exploring complete PES, this approach has to be ubiquitously used.

The electronic energy gap problem (whether it is related to optical excitation, emission, excitation transfer, etc.) involves two electronic states. The energy of both states (*E*_0_, initial, and *E*_1_, final) are described within the second-order approach as a function of the nuclei coordinates (**q**):
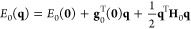
1
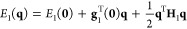
2where the expansion can be performed taking any structure as reference (denoted by **0** vector). The gradient vectors evaluated at the reference expansion point **0** are given by **g**_0_ and **g**_1_ for the initial and final states, respectively. Similarly, the Hessian matrices evaluated in **0** are **H**_0_ and **H**_1_. Finally, the transpose vector is indicated in all cases by superscript “T”.

The energy gap between states “0” and “1” is therefore
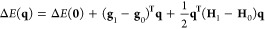
3where Δ*E*(**0**) ≡ *E*_1_(**0**) – *E*_0_(**0**), the variation of the energy gap defined as
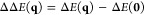
4

#### 3.1.2 Application of External Forces

When a mechanical force is applied to a molecular system, its structure changes, accommodating the nucleus position to a new equilibrium state where the internal and external forces cancel out. This process takes place in a defined adiabatic electronic state.

For instance, if a 0 → 1 excitation process is being considered, the forces are applied when the molecular system is in the initial state (i.e., “0” state), and the magnitude to be mechanically tuned is the excitation energy:
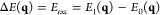
5The opposite situation holds when dealing with the emission energy from an excited state. Therefore, in this case, the new equilibrium structure in the electronic state “0” under the action of some external mechanical forces (**F**_*ext*_) fulfills the following condition:
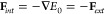
6Without loss of generality, we will consider in the following the initial state “0” as the state where the mechanical forces are being applied.

For the specific case where the forces are determined from a second-order expansion around the minimum of the initial state “0”, the above expression (eq 5) establishes the relation between the new equilibrium structure and the applied external force:
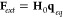
7

#### 3.1.3 Optimal Forces Varying the Energy Gap

If we consider the application of small forces, the displacements regarding the equilibrium structure will be small, and therefore, the PES in the initial “0” state can be approximated quadratically by eq 1 where the gradient term is zero:
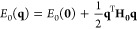
8

As mentioned above (eq 5), for the new equilibrium structure under the action of the external forces, the internal and external forces are opposed. Therefore, by solving eq 7, the new equilibrium structure is obtained:
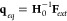
9In a more general context, this expansion can be made at any given point of the PES:
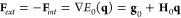
10where the magnitude of the applied force is therefore
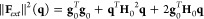
11Accordingly, the equilibrium structure is given in this general case by
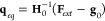
12It becomes apparent that the application of an external force will affect the equilibrium structure of the system and consequently (see eq 4) the energy gap variation between any two electronic states.

Among all the possible forces that can be applied, provoking a specific variation of the energy gap, there is usually only one optimal solution where the magnitude of the applied forces is minimal.

To determine the optimal forces, constrained optimization can be performed. Our implementation looks for the optimization of the energy difference (Δ***E*** (**q**), restrained for a constant force magnitude, ∥***F***_*ext*_∥^2^.

Using the Lagrange multipliers method, the following Lagrangian function is optimized:
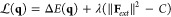
13where *C* stands for a constant (i.e., the square of the external force magnitude). By imposing , the following condition for the optimal force is obtained (see SI for more information):
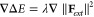
14
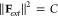
15where λ is the Lagrange.

**Figure 1:**
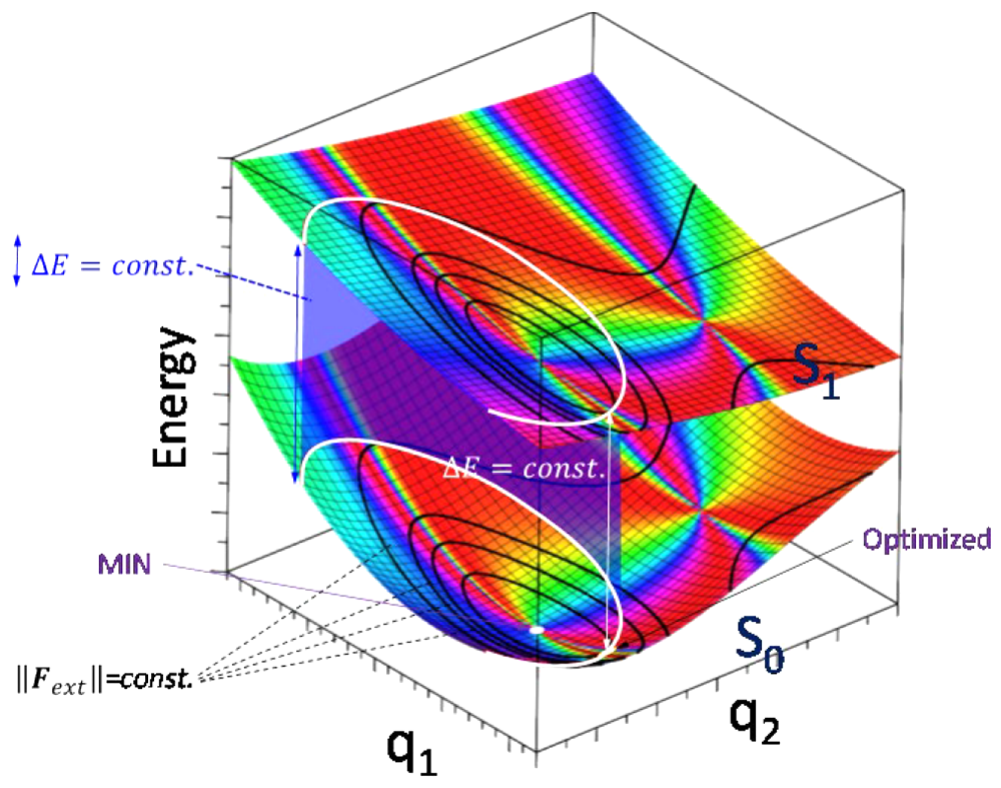
Schematic PES showing the location of optimized points according to eqs 14 and 15. Two PESs (e.g., S_0_ and S_1_) are represented as a function of two internal coordinates (i.e., q_1_ and q_2_). The surfaces are color-mapped with the value of the cosine angles formed by **∇**Δ*E* and **∇**∥***F*_*ext*_**∥^**2**^ vectors. Few curves of constant ∥***F***_*ext*_∥ (black) and Δ***E*** = *constant* (withe) are represented. For the optimized points, like the indicated, both isovalue curves become tangent.

Figure 1 shows the optimization procedure in model PES, where the optimization in the constrained curve (i.e., with constant ∥***F***_*ext*_∥) is reached where vectors **∇**Δ*E* and **∇**∥***F***_*ext*_∥^2^ become parallel.

For the specific case of small forces, it is possible to consider the quadratic approach of the PES around the minimum. Then, the optimal external force vector is equal to^25,26^
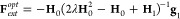
16Substituting into eq 6, the corresponding equilibrium structure is
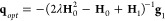
17Nevertheless, this approach may be not valid in the case of large external forces or forces applied along low-frequency normal modes, where a simple second-order approach of the PES can be insufficient to correctly predict the new equilibrium structures as well as the optimal forces tuning the energy gap.

The optimal forces represent a theoretical limit of the maximal mechanical response of the system. Nevertheless, this force is far from being directly applicable experimentally. Most of the experimental setups rely on the idea of applying force-pairs, as is the case of polymer embedded mechanophores^2^ or molecular force probes.^7^ Since any force can be decomposed in terms of multiple force-pairs,^25^ a possible approach to the optimal force could involve considering several force-pairs (i.e., the most contributing force-pairs). The quality of the resulting force will be in any case system dependent.

Additionally, under some assumptions, the substituent effect can be considered as an external force.^30^ In this case, the force pattern induced by the substituent is more complex than a simple force pair and can eventually match to some degree the optimal force. Therefore, inclusion of substituents can be also a strategy to achieve mechanical control of the energy gap.

#### 3.1.4 Optimal Forces for Local (Second-Order) Approach in the PES

Considering a second-order approach in the PES, the optimal external forces and the corresponding optimal minimum energy structures can be obtained from eqs 16 and 17. Determining the optimal structures implies to solve the linear equation system:
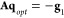
18where **A** = (2λ**H**_0_^2^ – **H**_0_ + **H**_1_)^−1^ is a symmetric square matrix.

It should be noted that, for some systems, the gradient **g**_1_ belongs to certain symmetry groups, so that if we express the variables of the system in symmetric coordinates, eq 18 can be expressed as
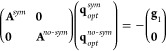
19where the superscript “*sym*” indicates symmetric coordinates (i.e., those totally symmetric), while “*no-sym*” corresponds to the non-totally symmetric coordinates. The gradient vector **g**_1_ belongs to a totally symmetric subspace. Thus, two systems of equations are obtained:
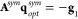
20
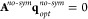
21By solving eq 20, for each value of λ, a unique solution for the optimal coordinates is obtained. These coordinates correspond to the symmetric subspace where the **g**_1_ vector belongs.

However, for the second system of equations (eq 21), the gradient component is 0; therefore, this yields an eigenvalue problem.

Equation 21 can be written in the form
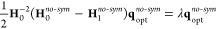
22Consequently, diagonalizing the matrix  the optimal coordinate solutions for the subspace in which the gradient has zero component, **q**_opt_^*no-symm*^, are obtained. The corresponding solutions (i.e., possible eigenvalues λ) are associated with the corresponding eigenvectors, which define the solutions for **q**_opt_^*no-symm*^. Therefore, for each eigenvalue λ of eq 21, it is possible to solve eq 20, obtaining the corresponding symmetric coordinates. The λ value consequently defines a displacement (i.e., optimal structural distortion as defined above). If this value corresponds to an eigenvalue of eq 21, there are infinite solutions for the nonsymmetric part and a finite number of solutions of the symmetric part (usually two meaningful solutions, one corresponding to an increase in the excitation energy and the other to the decrease). However, if λ is not equal to one of the eigenvalues, then only the nonsymmetric part has a component.

In a more general context, it is possible for any molecular system with a given symmetry to use symmetric coordinates in the expansion of the PESs. In these cases, it is possible to separate eq 18 into a subset of algebraic elements according to symmetry and thus separate the problem as described above. Nevertheless, in the following we will focus on the case of nonsymmetric constraints, valid as a general case.

#### 3.1.5 Optimal Mechanical Forces Exploring Complete PES

A local second-order approach of the PES may permit prediction of maximum variation of the energy gap for a given mechanical force magnitude if the displacement is relatively small (see discussion above). In order to overcome this limitation, it is necessary to explore complete PES and the use of an iterative algorithm locating optimal points (i.e., points of maximal variation of the energy gap for a constant force magnitude).

Summarizing, the LGMF algorithm considers a mechanical force applied on the system in the initial, i.e., the “0”, electronic state and looks for an optimal variation of the energy gap between states “1” and “0”. The steps are the following:

*Step 1*. The algorithm search starts at the **q**_0_ structure, which corresponds to an optimized point (e.g. the minimum energy point on the initial state). This point is characterized by a pair of optimal values: the external force magnitude (∥**F**_ext_(**q**_0_)∥) and the corresponding energy gap (Δ*E*(**q**_0_)).

**Figure 2:**
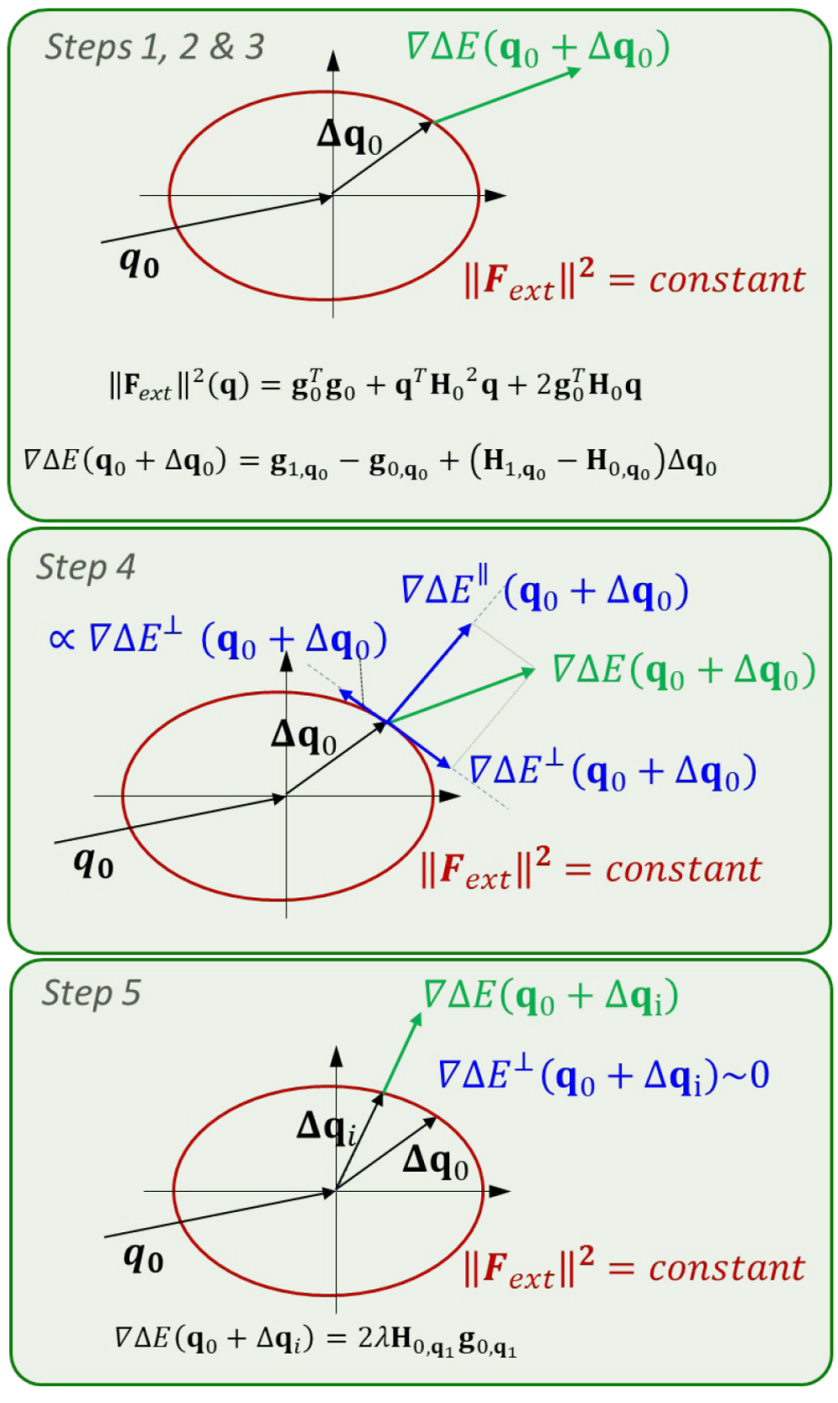
Schematic representation of the 5 different steps of the proposed LGMF algorithm. (i) *Steps 1*, *2*, and *3*: Expansion of PES locally on **q**_0_ up to second-order and performing a displacement **q**_1_ = **q**_0_ + Δ**q**_0_ with restricted external force ∥***F***_*ext*_∥^2^ = *constant*. (ii) Step 4: Decomposition of the **∇**Δ*E* vector in perpendicular (**∇**Δ*E*^⊥^) and parallel (**∇**Δ*E*^∥^) components to the ∥***F***_*ext*_∥^2^ = *constant* surface. (iii) Step 5: After convergence of step 4, the optimized point **q**_1_ = **q**_0_ + Δ**q**_*i*_ is determined. For this geometry, the condition **∇**Δ*E*^⊥^(**q**_0_ + Δ**q**_*i*_) ∼ 0 is fulfilled. The new structure, **q**_1_, is the origin for the new local expansion of the PESs starting at step 1.

A second-order approach around the **q**_0_ structure for the initial state is performed, which permits calculation of the energy gradient vector in the vicinity of this point, which equals the external force vector for the equilibrium structure (eq 5):
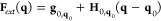
23where **H**_0,**q**_0__ and **g**_0,**q**_1__ are the Hessian matrix and gradient vector determined at the **q**_0_ structure in the initial electronic state (0). See Figure 2.

*Step 2*. The increase in the applied force magnitude between two optimized points is defined: Δ∥**F**_ext_∥. This value will be a constant for all the optimization processes and constitutes the control parameter. Δ∥**F**_ext_∥ should be chosen to ensure that the second-order approach will be valid in the vicinity of the expansion point, so in general, will fall in the order of tens of hundreds of pN, i.e., 10^–12^ Newtons.

A displacement from the **q**_0_ structure is performed along a given vector (this vector corresponds to the vector of the previous optimized point and will be the energy difference gradient vector if we are optimizing the first point). The displacement vector, Δ**q**_0_, is scaled in order to reach the condition Δ∥**F**_ext_∥ = *constant*. See Figure 2.

*Step 3*. For the new structure **q**_0_ + Δ**q**_0_, the energy gradient vector for both states (i.e., initial and final) is approximated by
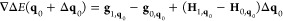
24where **g**_*i*,**q**_0__ indicates the gradient vector determined for the “*i*” state at **q**_0_ geometry. Additionally, the gradient of the force vector square is determined for this point by using the expansion made in **q**_0_:
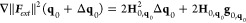
25If the predicted point is optimal, then eq 14 should be fulfilled within a convergence criterion (see Supporting Information for details). In other words, the **∇**Δ*E*(**q**_0_ + Δ**q**_0_) vector has to be parallel to the gradient of the square of the external force vector, so eq 14 is fulfilled for the current geometry:
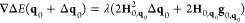
26If an optimal point is found, then the new optimal point **q**_1_ is **q**_1_ = **q**_0_ + Δ**q**_0_. See Figure 2.

And the optimization process continues at *step 5*. If not, the optimization continues in *step 4*.

*Step 4*. Taking the gradient vectors determined at **q**_0_ + Δ**q**_0_ geometry obtained from eq 26, the **∇**Δ*E*(**q**_0_ + Δ**q**_0_) vector is decomposed into two orthogonal vectors, one parallel to the **∇**∥***F***_*ext*_∥^2^ vector and the second orthogonal to it:
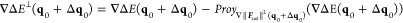
27
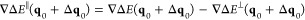
28The projection vectors are determined by computing the corresponding scalar products.

Since eq 14 ultimately implies that **∇**Δ*E*^⊥^(**q**_0_ + Δ**q**_0_) = 0, this component is reduced by performing a displacement along the **∇**Δ*E*^⊥^(**q**_0_ + Δ**q**_0_) vector (this displacement depends on a proportionality constant that is chosen on the basis of the validity of the first order approach in the Δ***E***_*exc*_ function; see Supporting Information for details). This displacement ensures that the magnitude of the external force vector remains constant. A new structure **q**_0_ + Δ**q**_1_ is obtained.

The optimization condition is again checked for the **q**_0_ + Δ**q**_1_ structure:
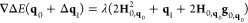
29If eq 29 is fulfilled, the optimization is finished, and **q**_1_ = **q**_0_ + Δ**q**_1_. In this case, the optimization continues at *step 5*; otherwise, this fourth step is repeated until convergence. See Figure 2.

*Step 5*. Once the new optimal geometry is determined in the converged iteration “*i*” of the *step 4*, **q**_1_ = **q**_0_ + Δ**q**_i_. The optimal solution is checked by determining the energy gradient (**g**_0,**q**_1__) and the Hessian matrix (**H**_0,**q**_1__) for this geometry:
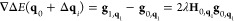
30If eq 30 is fulfilled, the optimized point **q**_1_ is confirmed and the algorithm process is continued in *step 1*. Note that in the previous step, the optimization condition is predicted on the basis of the validity of **g**_0,**q**_0__ and **H**_0,**q**_0__ in the vicinity of **q**_0_, but now the optimization condition is checked with exact gradient and Hessian for the optimal geometry, **q**_1_. If the condition is not fulfilled, the optimization process is continued in *step 3* using the new gradient and Hessian. See Figure 2.

##### 3.1.5.1 Multiple Solutions

Usually, the optimization process starts at the minimum energy structure of the initial state. From this point, there are, at least, two possible optimal solutions, one of them increasing and the other decreasing the energy gap, Δ*E*. This situation is general, and unless exceptional situations due to symmetry constraints, from each optimized point there are at least the above-mentioned two different optimal solutions for energy gap variation. Both solutions can be searched for by using opposite Δ**q** vectors, one of them in the positive direction of the Δ*E* vector (i.e., increasing energy gap) and the other one in the opposite direction (i.e., decreasing energy gap).

##### 3.1.5.2 Convergence Criteria

As discussed above, an optimal point is found when the vector **∇**Δ*E*_*exc*_^⊥^ vanishes. Additionally, the displacement vector between two consecutive points of the optimization, e.g., **q**_0_ + Δ**q**_i_ and **q**_0_ + Δ**q**_i+1_, tends to zero. For an optimized point, the angle formed by the vectors **∇**Δ***E*** and **∇**∥***F***_*ext*_∥^2^ is 0° or 180°, i.e., the cosine of the angle formed between both vectors, α, is equal to 1 or −1°. From a practical point of view, the numerical criterion for convergence is established between cos(α) = ±0.999 to cos(α) = ±0.99.

Additionally, the modulus of the force vector must be controlled. Usually the second-order approach in the initial state is enough to obtain the correct predictions. This is controlled by imposing, as discussed above, a small increase in the external force magnitude between consecutive points. The modulus of the external force vector is in fact a control parameter, so it is not strictly necessary to achieve a given value but to determine an optimal point. Nevertheless, in order to control the expected variation of the external force modulus, during the optimization process, only deviations up to 5% in the force strength are permitted. If an optimal point is found with larger deviation of the external force, the constant external force condition (see *step 2*) is recalculated with the updated information on the PES and the process starts again from this point.

##### 3.1.5.3 Hessian Update

In *step 5* of the LGMF algorithm, the energy gradient vector and the Hessian matrix are determined for the new structure (i.e., **q**_0_ + Δ**q**_1_). Usually, the gradient is determined analytically. Nevertheless, analytical calculation of Hessian matrix is significantly time-consuming. In this case, there are certain methods for updating the Hessian matrix using the displacement information Δ**q**_0_ and the gradient difference between two consecutively optimized points: **g**_0,**q**_1__ – **g**_0,**q**_0__. These methods are known as Quasi-Newton methods. One of the most widely used methods is the Broyden–Fletcher–Goldfarb–Shanno (BFGS) method.^29^ We have implemented this method for the Hessian update:
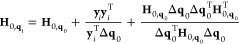
31where **y**_i_ = **g**_0,**q**_1__ – **g**_0,**q**_1__.

### 3.2 Algorithm Implementation

Here we apply the proposed LGMF algorithm to the study of the mechanical energy gap modulation of different molecular systems. The results are compared to those of existing mechanochemical models.

As anticipated in the discussion of the algorithm, this method is especially useful when dealing with low force constants, where the error at very small forces can be significant.

The necessity of exploring PESs for describing mechanochemical control of energy gap in molecular systems is specially related to the application of external forces with a significant component of low-frequency modes. For rigid systems, the second-order approach is usually valid for a wide range of forces, e.g., up to 2 nN.^25^

However, when dealing with very flexible systems, the above-mentioned force range implies very large displacements, and the surfaces are no longer representative of the behavior of the system. Another problem that arises in these cases is the coupling of low- and high-frequency modes along the mechanical coordinate, i.e., the coordinate associated with the application of an external force. From a practical point of view, this coupling is a source of numerical error when using Cartesian coordinates. Displacements in Cartesian coordinates do not correspond strictly to displacements along normal coordinates. Therefore, a given displacement along a high-frequency normal mode can provoke a small error in the energy prediction, but if it is coupled with some low-frequency mode, it can yield significant energy error.

Of course, this algorithm overcomes the intrinsic limit of the second-order approach, permitting the application of any kind of force strength and the real effect on complex PES. This allows the description, for instance, of the potential appearance of new minima or transition states, even when considering small external forces, as could be the case of energetically accessible conformations.^18^

#### 3.2.1 H_2_O, a Rigid Molecular System

As a first case-study, we chose a water molecule due to its small number of degrees of freedom and its rather rigid structure. The absence of low-frequency modes makes it a good example of a molecule where second-order approach is enough to describe large external forces up to few nN.

Water has a plane of symmetry that contains all of the atoms of the molecule. Moreover, the molecule will remain planar independently on the pair of forces, or a combination of them, applied to it. Therefore, we can apply the model described in Section 3.1.4 and use the symmetric coordinates by making the corresponding change of coordinates and rearranging them.

Let us define the molecule in the “xy” plane. We will consider two electronic states, the singlet ground-state and the first singlet excited state, labeled as “0” and “1”, respectively. Using second-order expansion of PES around the ground-state minimum it is possible to determine the optimal forces by solving eq 20.

The “z” subspace obviously corresponds to rotational and translational motions; therefore, the only valid solution for this subset of coordinates is **q**_*opt*_^*z*^ = **0**.

**Figure 3:**
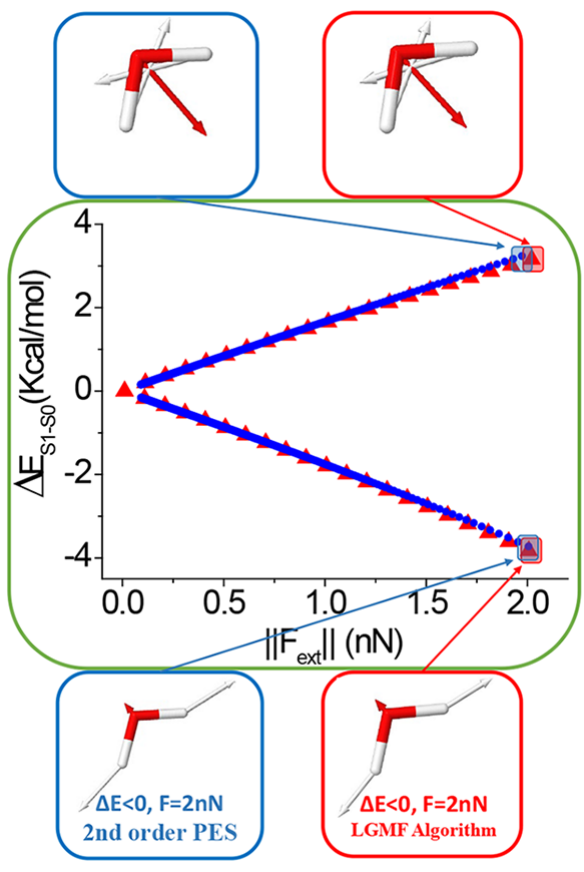
Optimal mechanical variation of the S_1_–S_0_ energy gap of the H_2_O molecule as a function of the applied external force magnitude. Blue circles correspond to the prediction based on a single second-order approach at the minimum energy structure, and red triangles to the LGMF algorithm exploring complete PES. Optimal force vectors are displayed for the indicated points using the second-order approach and the developed algorithm (vectors are colored according to atom type).

There are two sets of solutions, one corresponding to minimal force and the largest increase in the energy gap and the other to the largest decrease in the energy gap (see Figure 3). Solving therefore eq 30 for different values of λ (i.e., from λ = −0.233 to λ = 0.175) the structures corresponding to the optimal forces are obtained (see Figure 3).

This system can also be studied with the described LGMF algorithm for largest energy gap variation with minimal external force. To define the initial search direction and find the two possible solutions, the gradient vector and its opposite have been used. The two sets of solutions are plotted in Figure 3. The algorithm permits choosing the force range in which the optimal displacements are calculated, going in this case up to 2 nN.

#### 3.2.2 The Case of S_0_ to S_1_ Excitation in Semibullvalene

The second case-study is the semibullvalene (SBV) molecule. Its photochemical, photophysical, and mechanochemical behavior has been described in the literature.^25^ In particular, we focus on the study of the mechanical modulation of the S_0_ to S_1_ excitation energy, which is situated at ca. 140 kcal/mol.^25^

**Figure 4:**
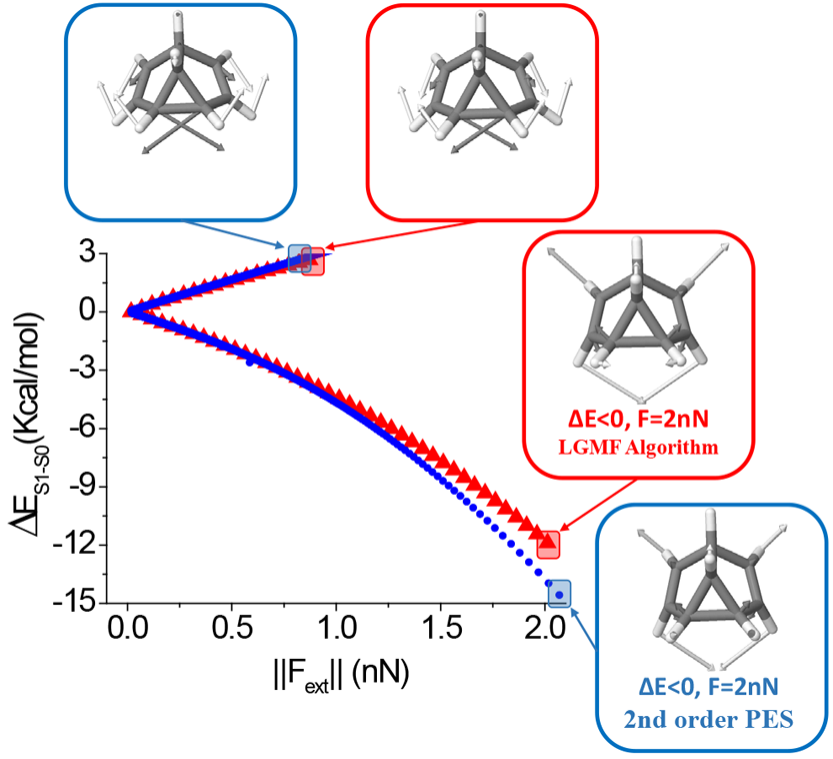
Optimal mechanical variation of the S_1_–S_0_ energy gap of the SBV molecule as a function of the applied external force magnitude. Blue circles correspond to the prediction based on a single second-order approach at the minimum energy structure. Red triangles correspond to the algorithm. Optimal force vectors are displayed for the indicated points using the second-order approach and the algorithm (vectors are colored according to atom type).

A previous mechanochemical study using eqs 16 and 17 predicted optimal external forces tuning the excitation energy. By scanning the λ multiplier, different optimal forces for increasing and decreasing the excitation energy were obtained up to 2 nN;^25^ see Figure 4.

In this case, the application of external forces may break the symmetry, and therefore, the problem cannot be split into two sets of equations as in the case of the water molecule.

Following a similar approach as in the case of the H_2_O molecule, the algorithm has been applied to the SBV molecule. Two branches of solutions corresponding to an increase and decrease in the excitation energy are obtained, being the mechanical response of *ca*. 3.1 and −5.9 kcal/(mol·nN), respectively (see Figure 4). Analogous to water molecules, the rigidity of the molecule makes the local second-order approach in the PES produce results similar to those when exploring PES with the implemented LGMF algorithm. Additionally, the optimal force vectors for increasing and decreasing the excitation energy are also similar using both methods; nevertheless, the deviation of the local second-order approach starts to be significant when the applied forces are larger than 1.5 nN approximately, where exploring complete PESs becomes necessary to achieve a correct prediction of the optimal external forces (see Figure 4).

Therefore, even in stiff molecules like SBV, the local second-order approach provides reasonable results for relatively low force magnitudes. However, exploring complete PESs becomes necessary when the force values are relatively high.

#### 3.2.3 The Pyrene Molecule

The third case-study is the S_0_ to S_1_ excitation of the pyrene molecule. There are several low-lying electronic states that are relevant to understand the absorption spectrum of the molecule;^26^ nevertheless, the S_0_ to S_1_ excitation is the first bright state, and we will focus on its mechanical modulation to test the developed algorithm. Pyrene is a planar molecule in which the in-plane normal modes correspond to relatively high force-constants. Nevertheless, out-of-plane modes have been associated in general low-frequency modes, with this system being an interesting molecule to test the necessity of exploring complete PESs to describe the mechanical modulation of the excitation energy. This modulation has been studied previously using single local second-order approximation for the ground and excited state according to eqs 20 and 21.^26^ This is possible due to the planarity of the molecule, where the separation between the in-plane and out-of-plane coordinates is possible, as explained above.

**Figure 5:**
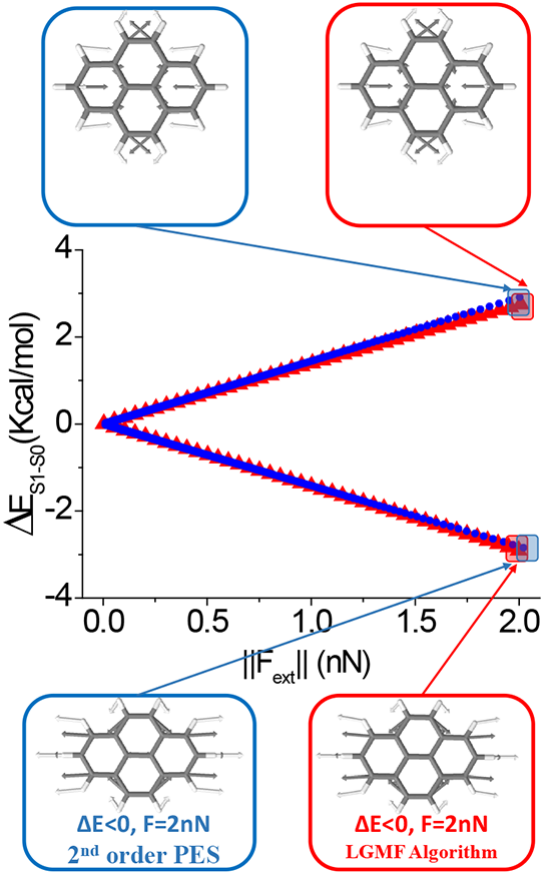
Optimal mechanical variation of the S_1_–S_0_ energy gap of pyrene for in-plane optimal external forces. Blue circles correspond to the prediction based on a single second-order approach at the minimum energy structure. Red triangles correspond to the LGMF algorithm. Optimal force vectors are displayed for the indicated points using the second-order approach and the algorithm (vectors are colored according to atom type).

The implementation of the algorithm in this case can also distinguishes between planar and out-of-plane forces. Using as the starting search direction that given by the excited state energy gradient vector (which is an in-plane vector), the two sets of solutions are found, i.e., those corresponding to the increase and decrease of the energy gap, respectively (see Figure 5).

The predictions related to geometry, mechanical response, and optimal force vectors are similar to both methodologies. This behavior is expected as the in-plane modes are, as discussed above, high frequency modes, and therefore a single local second-order approach is enough to describe the application of relatively large external forces, expanding this range up to ca. 2 nN (see Figure 5).

**Figure 6:**
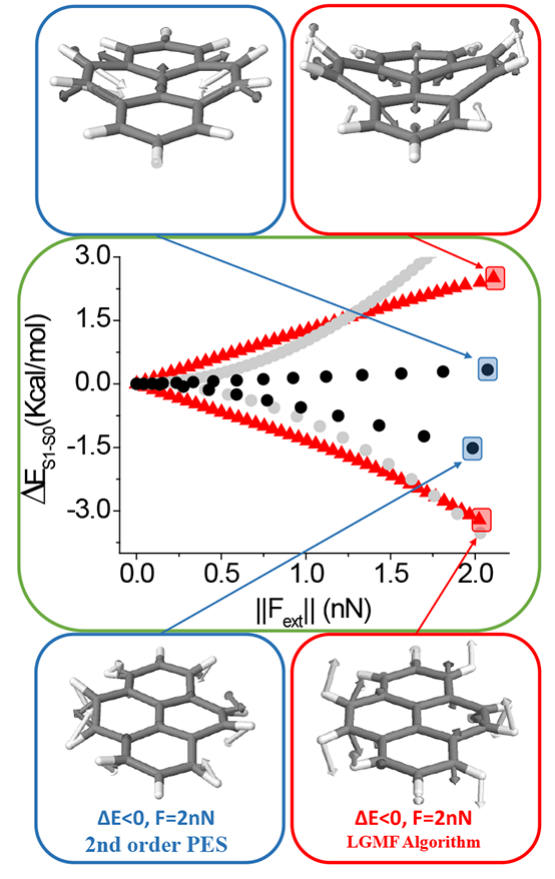
Optimal mechanical variation of the S_1_–S_0_ energy gap of pyrene for out-of-plane motions. Gray circles correspond to the prediction based on an analytical second-order approach at the minimum energy structure, while black circles correspond to the DFT corrected calculation (a large numerical error is detected here). Red triangles correspond to the exact solution determined with the LGMF algorithm. Optimal force vectors are displayed for the indicated points using the second-order approach and the algorithm (vectors are colored according to atom type).

By solving eq 22, a set of eigenvalues and eigenvectors are obtained, corresponding to the mechanical response of the excitation energy and the optimal mechanically induced displacements, respectively. In general, all these solutions associated with out-of-plane distortions correspond to relatively low-frequency modes (*ca*. 100 to 800 cm^–1^). In the following, we focus our discussion on the two solutions corresponding to the largest variation (positive and negative) of the excitation energy. The predictions made on the basis of the second-order approach of the PES are in general inefficient because of the problems already discussed (i.e., the validity of the second-order approach for large displacements along low-frequency modes and the high–low-frequency modes coupling). This becomes apparent when the predicted energies and forces are compared with exactly calculated energies (see Figure 6).

This deviation is relatively low in the prediction of excitation energy variation but is very large in the prediction of the forces (see Figure 6). The reason for that relies in the fact that force calculation only depends on the ground-state, but the excitation energy, which corresponds to the energy difference between both PESs, make in some way the above-mentioned errors cancel out, providing more realistic predictions. In any case, only the optimal displacements are qualitatively similar to those obtained when considering complete PESs.

Applying the LGMF algorithm, the correct mechanical response of the excitation energy is obtained. In this case, all of the points are optimal solutions within the convergence criteria discussed above. The two sets of solutions (i.e., increase and decrease of the excitation energy) are determined, showing a mechanical response of ca. 1.5 kcal·mol^–1^·nN^–1^ (see Figure 6).

## 4 Conclusions

We have developed an algorithm for the exact determination of the optimal mechanical response in the energy gap variation between two electronic states by exploring the PESs. This algorithm explores the PES by imposing a restriction on the magnitude of the applied force, determining the force provoking the largest variation in the energy gap between the two states. By iteratively increasing the force magnitude, different solutions (increasing and decreasing the energy gap) are found.

The solutions obtained with the LGMF algorithm are exact depending only on the convergence criteria applied for the calculation. The algorithm overcomes the intrinsic limitations of previous proposed methods using the single second-order approach of the PESs (i.e., the validity of this approach for large displacements and the coupling between low- and high-frequency modes).

The LGMF algorithm has been implemented for several molecular systems showing a different range of flexibility. The single second-order approach of PES fails in predicting optimal forces modulating the energy gap for (*i*) large force magnitudes even in stiff molecules and (*ii*) small forces when low-frequency modes are involved in the optimal solutions.

The developed algorithm provides an exact solution for any molecular system using energy gradient vectors and Hessian matrices for the two electronic states. The LGMF algorithm is available at: https://github.com/resmol/Optimal-Force.git

## Supporting Information Available

Details of the minimization procedure and Hessian update, algorithm convergence criteria, and Cartesian coordinates of relevant structures (PDF)
