# Supplementary material for: An Algorithm Predicting the Optimal Mechanical Response of Electronic Energy Difference
Source: J Chem Theory Comput. 2023 Sep 5;19(18):6392–401. doi: 10.1021/acs.jctc.3c00490 (PMC10536970; doi:10.1021/acs.jctc.3c00490)
Supplement: Supplementary file 1 — ct3c00490_si_001.pdf [file ct3c00490_si_001.pdf]

Supporting Information

An algorithm predicting the optimal mechanical response of electronic energy difference

Alejandro Jodra,[a,] Cristina García-Iriepa[a,b] and Luis Manuel Frutos[a,b,*]*

[a] Universidad de Alcalá, Departamento de Química Analítica, Química Física e Ingeniería Química, Grupo de Reactividad y Estructura Molecular (RESMOL), Alcalá de Henares 28806 Madrid, Spain; Instituto de Investigación Química ''Andrés M. del Río'' (IQAR), Universidad de Alcalá, Alcalá de Henares 28806 Madrid, Spain.

[b] Universidad de Alcalá, Departamento de Química Analítica, Química Física e Ingeniería Química, Grupo de Reactividad y Estructura Molecular (RESMOL), Alcalá de Henares 28806 Madrid, Spain.

Content

1. Minimal search and Hessian update

2. Algorithm convergence criteria

3. Cartesian Coordinates of relevant structures.

1. Minimal search and Hessian update

The algorithm developed in this study deals with the general problem of finding critical points for a differentiable scalar function, which is the case of a potential energy surfaces for a given electronic state or any combination of them. The most commonly used methods for finding these characteristic points are based on Newton's methods. The aim of these methods is to search for the zeros of a given function. For the stationary points, it is assumed that the gradient at that point is 0 within a convergence threshold.

Let us assume a scalar function $g(\mathbf{x})$ where $\mathbf{x} \in \mathbb{R}^n$. For determining the zeros of this function, we use the secant method. To do this, the function is expanded at the point $\mathbf{x_0}$ using the Taylor polynomial of first order.

$$g(\mathbf{x}) \approx g(\mathbf{x_0}) + \mathbf{J}(\mathbf{x_0})(\mathbf{x} - \mathbf{x_0}) \qquad \text{(S1)}$$

where $\mathbf{J}(\mathbf{x_0})$ is the Jacobian matrix that will contain the first derivatives.

$$\mathbf{J}(\mathbf{x_0}) = \begin{pmatrix} \dfrac{\partial g(\mathbf{x_0})}{\partial x_1} \\ \vdots \\ \dfrac{\partial g(\mathbf{x_0})}{\partial x_n} \end{pmatrix}$$

Known the $g(\mathbf{x})$ function, we look for the point that satisfies $g(\mathbf{x}) = 0$.

$$0 = g(\mathbf{x_0}) + \mathbf{J}(\mathbf{x_0})(\mathbf{x} - \mathbf{x_0}) \quad \text{(S2)}$$

By solving, we get:

$$\mathbf{x} = \mathbf{x_0} - [\mathbf{J}(\mathbf{x_0})]^{-1} g(\mathbf{x_0}) \quad \text{(S3)}$$

In this way we obtain the value of the zero of the function. However, since we do not know the exact function (we have approximated it using a Taylor polynomial of order 1, see Eq. S1), it is necessary to check if this point is really a zero of the function. To do this, it is necessary to evaluate the function at the new point obtained using Eq. S1 and re-compute the point where $g(\mathbf{x}) = 0$ is fulfilled. Therefore, by iterating according to this procedure, a sequential approach to the real solution is fulfilled. Iteratively, the zero of the function is obtained:

$$\mathbf{x_{i+1}} = \mathbf{x_i} - [\mathbf{J}(\mathbf{x}_i)]^{-1} g(\mathbf{x}_i) \quad \text{(S4)}$$

A zero of the function is obtained when $\|\mathbf{x_{i+1}} - \mathbf{x_i}\| < C$, where C is a critical value that ensures the convergence of the procedure.

The search for critical points is based on the same principle. Let's assume a scalar function $f(\mathbf{x})$ as above. It is assumed that the function can be locally approximated to a quadratic function.

$$f(\mathbf{x_i} + \Delta\mathbf{x}_i) \approx f(\mathbf{x}_i) + \nabla f(\mathbf{x}_i)\Delta\mathbf{x}_i + \frac{1}{2}\Delta\mathbf{x}_i{}^T \mathbf{B}(\mathbf{x}_i)\Delta\mathbf{x}_i \quad \text{(S5)}$$

where for convenience we have imposed $\Delta\mathbf{x}_i = \mathbf{x_{i+1}} - \mathbf{x_i}$ y $\nabla f = \mathbf{J}(\mathbf{x}_i)$. In addition $\mathbf{B}(\mathbf{x}_i)$ is the Hessian matrix

$$\mathbf{B}(\mathbf{x}_i) = \begin{pmatrix} \frac{\partial f(\mathbf{x}_i)}{\partial x_1 x_1} & \cdots & \frac{\partial f(\mathbf{x}_i)}{\partial x_1 x_n} \\ \vdots & \ddots & \vdots \\ \frac{\partial f(\mathbf{x}_i)}{\partial x_n x_1} & \cdots & \frac{\partial f(\mathbf{x}_i)}{\partial x_n x_n} \end{pmatrix}$$

As has been noted above, at the critical point it is satisfied that the gradient vanishes. Therefore, by deriving Eq. S5

$$\nabla f(\mathbf{x}_i + \Delta\mathbf{x}_i) = \nabla f(\mathbf{x}_i) + \mathbf{B}(\mathbf{x}_i)\Delta\mathbf{x}_i \quad \text{(S6)}$$

Equating Eq. 6 give rise to:

$$\Delta\mathbf{x}_i = -[\mathbf{B}(\mathbf{x}_i)]^{-1}\nabla f(\mathbf{x}_i) \quad \text{(S7)}$$

As can be seen the equation is similar to Eq. S4. The difference is that in this case we are looking for the zeros of a vector function $g: \mathbb{R}^{\boldsymbol{n}} \rightarrow \mathbb{R}^{\boldsymbol{n}}$ instead of a scalar function $g: \mathbb{R}^{\boldsymbol{n}} \rightarrow \mathbb{R}$**.** So instead of having the matrix $\mathbf{J}(\mathbf{x}_i)$ of (1xn) dimension, we have the matrix $\mathbf{B}(\mathbf{x}_i)$ of (nxn) dimension. So, iteratively using Eq. S7, we will obtain the critical point of the function.

Newton's method, however, can be included in a group of optimisation methods known as linear search methods. In this group, the search for the minimum is carried out by performing a displacement known as the descent direction, which minimises the value of the function, converging to the minimum.

$$\Delta\mathbf{x}_i = \alpha_i \mathbf{p}(\mathbf{x}_i) \quad \text{(S8)}$$

where $\mathbf{p}(\mathbf{x}_i)$ is the search vector or direction of descent and $\alpha_i \in \mathbb{R}_+$ is a constant known as the step length, which scales the displacement vector in order to ensure certain conditions and accelerate convergence. Depending on the descent vector and the step length constant, α_i, we can obtain different optimisation methods.

As shown in Fig. 2, for each of the iteration steps it is necessary to calculate both the gradient and the Hessian matrix. In cases where the calculation of the Hessian matrix is too time-consuming, there are certain methods for updating the Hessian matrix using the displacement and

gradient difference information. $\Delta\mathbf{x}_{i-1}$ and gradient difference information $\nabla f(\mathbf{x}_i) - \nabla f(\mathbf{x}_{i-1})$. These methods are known as Quasi-Newton methods. One of the most widely used methods is the Broyden-Fletcher-Goldfarb-Shanno (BFGS) method [S1]. It ensures that the updated Hessian is a positive definite matrix and is computed as

$$\mathbf{B}_{i+1} = \mathbf{B}_i + \frac{\mathbf{y}_i\mathbf{y}_i^T}{\mathbf{y}_i^T\mathbf{s}_i} + \frac{\mathbf{B}_i\mathbf{s}_i\mathbf{s}_i^T\mathbf{B}_i^T}{\mathbf{s}_i^T\mathbf{B}_i\mathbf{s}_i} \qquad \text{(S9)}$$

where $\mathbf{y}_i = \nabla f(\mathbf{x}_{i+1}) - \nabla f(\mathbf{x}_i)$ y $\mathbf{s}_i = \Delta\mathbf{x}_i$. According to Eq. S9, for the update to be possible and the process to yield a positive definite matrix $\mathbf{B}_{i+1}$ defined positive matrix, the curvature condition must be $\mathbf{s}_i^T\mathbf{y}_i > \mathbf{0}$. If necessary, to ensure the correct update of the Hessian, we can force the displacement to meet Wolfe's conditions [S1]. To ensure that these conditions are met, the optimisation is performed using the equation

$$\Delta\mathbf{x}_i = -\alpha_i[\mathbf{B}(\mathbf{x}_i)]^{-1}\nabla f(\mathbf{x}_i) \qquad \text{(S10)}$$

where α_i allows us to scale the vector to ensure that the conditions are optimal for the Hessian update.

It can be shown that these conditions are satisfied for convex or locally convex functions. In the method proposed in this article, although we do not do an unconstrained optimisation leading to the minimum of the function, throughout the constrained optimisation, the points are located always around a minimum of a scalar function (PES). Near such minima the second-order approximations are adequate: such second-order surfaces fulfil the condition of convex functions. Therefore, the BFGS method is a good method for updating the Hessian for displacements along local minima of PESs. In order to check how good the Hessian update is compared to the exact Hessian, we performed the computation of optimal forces using both. These results can be seen in figure S1.

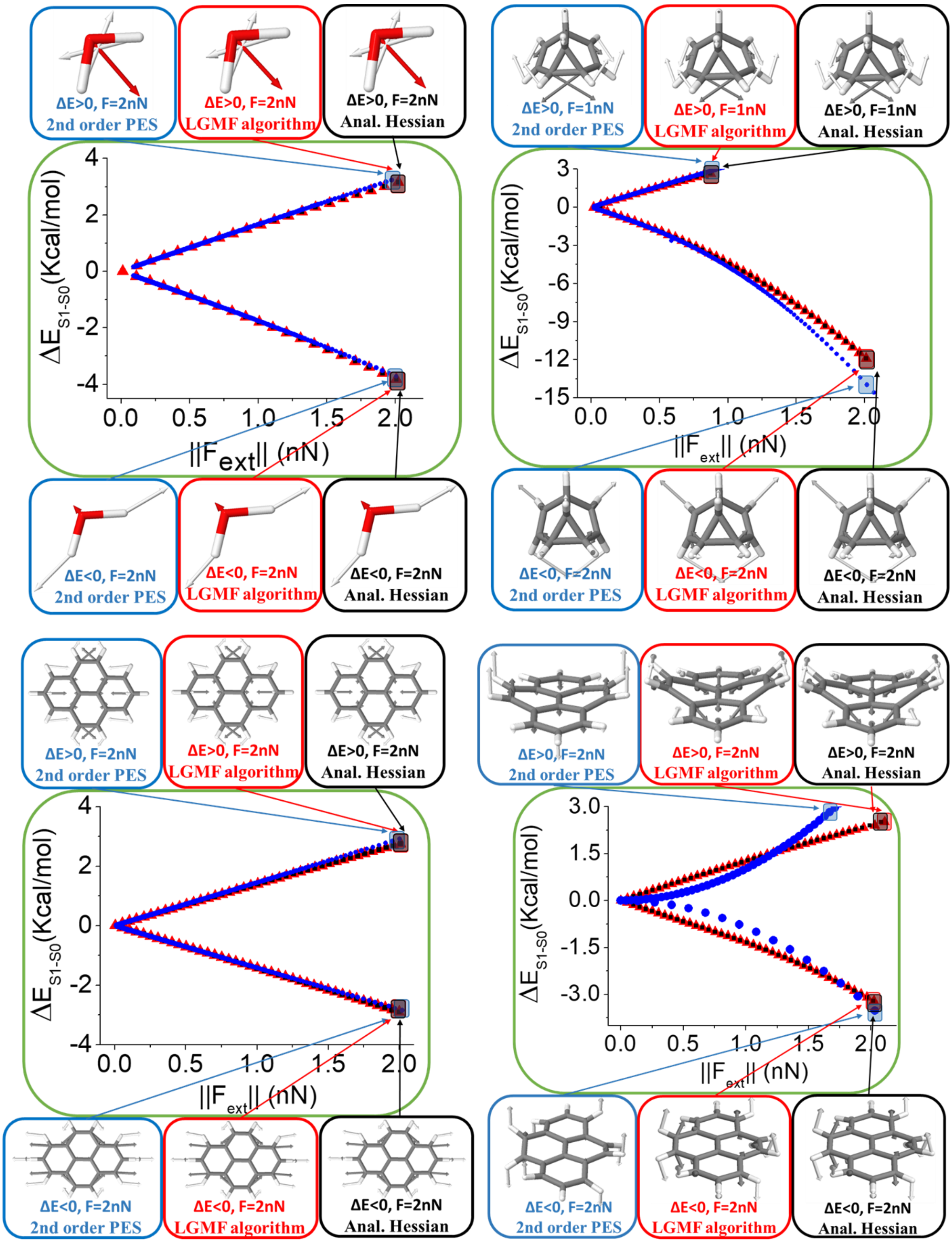

Figure S1. Predictions made with the LGMF algorithm using updated hessians (red points) and analytical hessians (black points) for the studied systems: (a) up-left: water molecule, (b) up-right: SBV system, (c) down-left: In-plane forces in pyrene (d) down-right: out-of-plane forces in pyrene. Blue dots obtained from single local approximation are shown for the sake of comparison.

2. Algorithm convergence criteria

The problem we address in this article is the conditional stationary point search problem. Solving these problems can be done with the Lagrange multiplier method, which states that a constrained problem of n variables gives rise to an unconstrained problem of n+k variables where k is the number of constraints. For a scalar function $f(\mathbf{x})$ where $\{\mathbf{x} \in \mathbb{R}^n\}$ and a constraint $g(\mathbf{x}) = c$, it can be shown that it is possible to construct a function $L(\boldsymbol{x}, \lambda) = f - \lambda g$ which will be a linear combination of the two above. The restrained stationary points fulfil $\boldsymbol{\nabla} L = \mathbf{0}$. It is possible to use the above method for searching for zeros and use Eq. S6 and S7 to iteratively find the solutions. However, the above condition implies that the solutions must satisfy

$$\boldsymbol{\nabla} f = \lambda \boldsymbol{\nabla} g \qquad \text{(S11)}$$

where λ is a scalar known as the Lagrange multiplier. Then, we will resort to linear search methods, making use of Eq. S8 to find those points that satisfy condition given by S11. In particular, as we discuss in the main text, the direction of displacement will be determined by the expression

$$\Delta \mathbf{q}_i = -\alpha_i \boldsymbol{\nabla} \Delta E_{exc}^{\perp}(\mathbf{q}_i) \qquad \text{(S12)}$$

The linear search methods following Eq. 8 are based on the fact that the direction of the displacement must minimize the function to be optimised. Therefore, they are methods used for the search of minima. But the actual problem is about finding conditional extrema, both minima and maxima. So to find local maxima, we must impose the opposite condition on the displacement vector, that is, maximizing the function. This means that, for the search for the local maximum, the displacement vector must be

$$\Delta \mathbf{q}_i = \alpha_i \boldsymbol{\nabla} \Delta E_{exc}^{\perp}(\mathbf{q}_i) \qquad \text{(S13)}$$

Once we have defined the displacement vector, the convergence have to be checked. By means of the iteratively linear search processes, different points that are sequentially closer to the

solution are found, reaching a point that is close to it within a certain threshold. Therefore, the solution has been reached when $||\mathbf{q}_{i+1} - \mathbf{q}_i|| < C$, where C is a critical value that ensures meaningful convergence criteria. The solutions satisfy the condition of conditional extremum $\nabla L = \mathbf{0}$ which implies that $\nabla \Delta E_{exc}^{\perp} = \mathbf{0}$. Therefore, it is possible to use as a convergence criteria: $||\nabla \Delta E_{exc}^{\perp}(\mathbf{q}_{i+1})|| < C$, where C is again a threshold value. $\nabla \Delta E_{exc}^{\perp}$ provides a measure of the angle between the vectors $\nabla \Delta E_{exc}$ and $\nabla ||\boldsymbol{F}_{ext}||^2$. It tends to 1, when $||\nabla \Delta E_{exc}^{\perp}(\mathbf{q}_{i+1})||$ becomes smaller enough. The convergence is reached when the cosine of the angle is close to 1.

In linear search methods, the choice of initial search direction is important, but also the modulus of the displacement. If the displacement is too small, convergence to the point will be slow. On the other hand, if it is too large, it does not converge to be solution, but rather provokes an oscillation along the desired point. This displacement is scaled by the constant α_i. There is a wide variety of methods for the calculation of the α_i which are based on the minimization of the function to be optimised at each step, in this case $min_{\alpha} L(\mathbf{q}_i - \alpha \nabla \Delta E_{exc}^{\perp}(\mathbf{q}_i))$[S1]. However, in this case, we implement a constant parameter $\alpha = 0.0035$ obtained by optimizing the efficiency of the algorithm in different tested molecular systems. The displacement then has been taken as:

$$\Delta \mathbf{q}_i = -0.0035 \nabla \Delta E_{exc}^{\perp}(\mathbf{q}_i) \qquad \text{(S14)}$$

$$\Delta \mathbf{q}_i = 0.0035 \nabla \Delta E_{exc}^{\perp}(\mathbf{q}_i) \qquad \text{(S15)}$$

3. Cartesian Coordinates of relevant structures

S_0 minimum H_2O

1	0.761443	-0.479181	0.000000
8	0.000000	0.119790	0.000000
1	-0.761443	-0.479136	0.000000

ΔE>0, F=2nN LGMF algorithm / **Force**

H	0.7432074	-0.4792010	0.0000000	-0.5399816	0.2635980	-0.0000000
O	0.0000143	0.1198590	-0.0000000	-0.0000073	-0.5271508	0.0000000
H	-0.7432216	-0.4791490	0.0000000	0.5399889	0.2635528	-0.0000000

ΔE<0, F=2nN LGMF algorithm / **Force**

H	0.7973797	-0.4712930	-0.0000000	0.6862084	-0.0984479	0.0000000
O	-0.0000077	0.1041288	0.0000000	0.0000412	0.1969426	-0.0000000
H	-0.7973719	-0.4713263	-0.0000000	-0.6862496	-0.0984947	-0.0000000

ΔE>0, F=2nN 2nd order PES / **Force**

1	0.7441014	-0.4788234	0.0000000	-1.3047198	0.6576236	-0.0000000
8	0.0000013	0.1190791	-0.0000000	0.0000810	-1.3151075	0.0000000
1	-0.7441027	-0.4787811	-0.0000000	1.3046388	0.6574839	-0.0000000

ΔE<0, F=2nN 2nd order PES / **Force**

1	0.7955139	-0.4715238	0.0000000	1.6791742	-0.2197542	0.0000000
8	-0.0000013	0.1044748	-0.0000000	-0.0001173	0.4393182	0.0000000
1	-0.7955126	-0.4714764	0.0000000	-1.6790570	-0.2195641	0.0000000

S_0 minimum SBV

6	1.179127	-1.106534	-0.127059
6	1.546654	0.052515	-0.683882
6	0.792254	1.180074	-0.101947
6	0.000224	0.633987	1.043430
6	-0.000455	-0.897151	0.815823
6	-1.180481	-1.105372	-0.126844
6	-0.790961	1.180774	-0.102189
6	-1.546309	0.053938	-0.684133
1	1.522534	-2.091185	-0.427193
1	2.262371	0.164238	-1.491405
1	1.234445	2.170424	-0.057680

1	0.000286	1.070941	2.036895
1	-0.000598	-1.515843	1.716543
1	-1.525165	-2.089691	-0.426641
1	-1.232360	2.171472	-0.057977
1	-2.261834	0.166253	-1.491737

	**ΔE>0, F=2nN LGMF algorithm**				**Force**	
C	1.2143375	-1.1167735	-0.1122037	0.1018621	0.1385274	-0.0518949
C	1.5532832	0.0379833	-0.6927872	-0.0288360	-0.1667329	0.1056119
C	0.7770939	1.1684005	-0.1276036	-0.3646870	0.1038578	-0.2137397
C	0.0027740	0.6296295	1.0257945	0.0007996	0.0459872	0.0483174
C	0.0041869	-0.9039436	0.7958409	0.0002747	0.0243308	0.0576223
C	-1.1740168	-1.0954409	-0.1509720	-0.1000332	0.1386654	-0.0526096
C	-0.7956377	1.1941499	-0.1180402	0.3642413	0.1040716	-0.2149012
C	-1.5733762	0.0695749	-0.6702611	0.0280045	-0.1651423	0.1061576
H	1.6177467	-2.0960100	-0.3637561	0.1148892	0.1702624	-0.2084553
H	2.3055739	0.1573075	-1.4646933	0.0915282	-0.1969479	0.1912477
H	1.2367128	2.1473280	-0.0662250	0.1345005	-0.1625828	0.1127452
H	0.0063732	1.0682351	2.0183985	-0.0006186	0.1133913	-0.0050671
H	-0.0372411	-1.5217111	1.6982004	-0.0006273	0.0385251	0.0298906
H	-1.5253016	-2.0664152	-0.4808133	-0.1127292	0.1708359	-0.2102746
H	-1.2410035	2.1847883	-0.0591451	-0.1353929	-0.1618352	0.1127228
H	-2.3717742	0.1817356	-1.3977239	-0.0931759	-0.1952138	0.1926269

	**ΔE<0, F=2nN LGMF algorithm**				**Force**	
C	1.1987854	-1.0939873	-0.1114327	0.0457112	-0.0121480	0.0292828
C	1.4686317	0.0609579	-0.7383683	-0.0481531	0.0530779	-0.1034494
C	0.7757820	1.1859901	-0.0734070	0.0915297	-0.0367039	0.0976899
C	0.0001922	0.6111874	1.0732843	0.0004499	-0.0486651	-0.0033099
C	-0.0002761	-0.9125259	0.8284238	0.0006516	-0.0376404	-0.0729963
C	-1.2000634	-1.0930571	-0.1105309	-0.0444190	-0.0120389	0.0296603
C	-0.7735810	1.1863649	-0.0746125	-0.0916052	-0.0364013	0.0959914
C	-1.4676330	0.0616197	-0.7390378	0.0485252	0.0533462	-0.1038430
H	1.6629855	-2.0600761	-0.2891924	0.2479499	0.0258362	0.3153077
H	1.9540511	0.1846933	-1.7044831	-0.4124376	0.0712350	-0.2958002
H	1.2361030	2.1679957	-0.0085223	-0.0850254	0.0558676	0.0332093

H	-0.0010374	1.0116327	2.0811797	-0.0016392	-0.1304802	0.0346641
H	-0.0001912	-1.5676749	1.7016435	0.0000984	-0.0990029	-0.1108777
H	-1.6673771	-2.0582188	-0.2851103	-0.2490574	0.0275647	0.3192002
H	-1.2333445	2.1687074	-0.0107650	0.0850805	0.0560878	0.0315009
H	-1.9532944	0.1852276	-1.7050496	0.4123406	0.0700652	-0.2962300
	**ΔE>0, F=2nN 2nd order PES**				**Force**	
6	1.1955264	-1.1042324	-0.1281878	0.1199269	0.1384538	-0.0368707
6	1.5613750	0.0522682	-0.6812346	-0.0402724	-0.1658912	0.0917431
6	0.7848192	1.1835463	-0.1240616	-0.4279437	0.1195878	-0.2701937
6	0.0002239	0.6317579	1.0252217	-0.0003152	0.0272656	0.0446308
6	-0.0004524	-0.9022187	0.7996652	0.0000026	0.0133731	0.0539343
6	-1.1969913	-1.1030749	-0.1278148	-0.1205707	0.1388767	-0.0363255
6	-0.7834693	1.1841933	-0.1243405	0.4283560	0.1190040	-0.2698863
6	-1.5609583	0.0536488	-0.6815130	0.0406973	-0.1660491	0.0919802
1	1.5803163	-2.0719811	-0.4340115	0.1633853	0.1830679	-0.2023051
1	2.3277602	0.1484052	-1.4444178	0.1189579	-0.1984265	0.2049523
1	1.2560851	2.1585091	-0.0429123	0.1639768	-0.1688057	0.1529728
1	0.0002615	1.0818149	2.0130825	0.0001420	0.1091554	-0.0143809
1	-0.0004432	-1.5132343	1.7058743	0.0006050	0.0348672	0.0341186
1	-1.5832416	-2.0704039	-0.4331851	-0.1640831	0.1836190	-0.2023323
1	-1.2540865	2.1594662	-0.0432223	-0.1647646	-0.1693874	0.1532774
1	-2.3269929	0.1503753	-1.4449378	-0.1181002	-0.1987108	0.2046849
	**ΔE<0, F=2nN 2nd order PES**				**Force**	
6	1.1712440	-1.0943735	-0.0980027	0.1106176	-0.0010781	0.1482904
6	1.4390125	0.0575897	-0.7416807	-0.1877193	0.0930703	-0.2676941
6	0.7760053	1.1922810	-0.0555269	0.1994292	-0.0473117	0.2864437
6	0.0002088	0.6222994	1.0952036	0.0004580	-0.0887973	0.0423442
6	-0.0004162	-0.9006837	0.8717307	0.0002752	-0.0422744	-0.0611080
6	-1.1725230	-1.0932220	-0.0977642	-0.1095088	-0.0008127	0.1480732
6	-0.7744794	1.1929360	-0.0559908	-0.1991032	-0.0468636	0.2849866
6	-1.4385092	0.0588248	-0.7419883	0.1878384	0.0931862	-0.2678437
1	1.5732996	-2.0780749	-0.3344225	0.4857220	0.0000564	0.6060935
1	1.8903197	0.1584823	-1.7865535	-1.0036933	0.0970655	-0.9728476
1	1.2529044	2.1668327	0.0230465	-0.1038695	0.1099806	0.2081785

1	-0.0000840	1.0431528	2.0953502	-0.0016236	-0.2294398	0.1163919
1	-0.0006126	-1.5385578	1.7584209	-0.0003153	-0.1440230	-0.1133084
1	-1.5760489	-2.0765162	-0.3335337	-0.4854354	0.0009675	0.6069355
1	-1.2505688	2.1678914	0.0222730	0.1046340	0.1103676	0.2065977
1	-1.8900202	0.1599779	-1.7865572	1.0022940	0.0959065	-0.9715334

S_0 minimum Pyrene in plane

6	-1.684758	-2.691987	0.000000
6	-1.709755	-1.286663	0.000000
6	-0.472782	-0.570536	0.000000
6	0.764090	-1.286839	0.000000
6	0.738893	-2.692159	0.000000
6	-0.472982	-3.383879	0.000000
6	-2.938510	-0.537999	0.000000
6	-2.938413	0.825196	0.000000
6	-1.709552	1.573685	0.000000
6	-0.472680	0.857382	0.000000
6	1.992951	-0.538350	0.000000
6	1.993048	0.824845	0.000000
6	0.764294	1.573509	0.000000
6	0.739296	2.978833	0.000000
6	-0.472480	3.670725	0.000000
6	-1.684355	2.979005	0.000000
1	-2.624031	-3.238864	0.000000
1	1.678089	-3.239170	0.000000
1	-0.473059	-4.470041	0.000000
1	-3.877083	-1.086244	0.000000
1	-3.876908	1.373575	0.000000
1	2.931446	-1.086729	0.000000
1	2.931622	1.373090	0.000000
1	1.678569	3.525711	0.000000
1	-0.472402	4.756887	0.000000
1	-2.623550	3.526016	0.000000

ΔE>0, F=2nN LGMF algorithm				Force		
C	-1.6858540	-2.6471862	-0.0000006	0.0201178	0.2590813	-0.0000059
C	-1.7163378	-1.2579668	0.0000027	0.0860993	-0.0087474	0.0000095
C	-0.4727416	-0.5617525	0.0000041	-0.0000098	-0.0774686	0.0000149
C	0.7707556	-1.2581438	0.0000000	-0.0860929	-0.0087253	-0.0000033
C	0.7400766	-2.6473585	-0.0000031	-0.0200671	0.2590843	-0.0000193
C	-0.4729371	-3.3287283	-0.0000021	0.0000140	0.2317467	-0.0000043
C	-2.9632128	-0.5325360	0.0000007	-0.1499550	0.1879637	-0.0000068
C	-2.9631159	0.8197145	-0.0000000	-0.1499887	-0.1879206	-0.0000103
C	-1.7161379	1.5449662	0.0000022	0.0861041	0.0087223	0.0000033
C	-0.4726413	0.8485747	0.0000041	0.0000077	0.0774831	0.0000124
C	2.0177335	-0.5328923	0.0000000	0.1499860	0.1879002	-0.0000124
C	2.0178292	0.8193583	-0.0000001	0.1499537	-0.1879380	0.0000016
C	0.7709545	1.5447885	-0.0000001	-0.0861048	0.0087492	-0.0000088
C	0.7404715	2.9340074	-0.0000017	-0.0201267	-0.2590805	-0.0000064
C	-0.4724445	3.6155502	0.0000000	-0.0000068	-0.2317391	0.0000050
C	-1.6854580	2.9341810	0.0000000	0.0200666	-0.2590742	0.0000016
H	-2.6317049	-3.1729036	-0.0000000	0.0419663	0.2046687	0.0000020
H	1.6858532	-3.1732095	0.0000000	-0.0419349	0.2046711	0.0000096
H	-0.4730119	-4.4074317	-0.0000000	0.0000253	0.2253362	0.0000060
H	-3.9065550	-1.0745476	-0.0000000	-0.0983607	0.0927657	0.0000013
H	-3.9063786	1.3618631	0.0000000	-0.0983523	-0.0927513	0.0000035
H	2.9609985	-1.0750374	0.0000041	0.0983654	0.0927622	0.0000090
H	2.9611716	1.3613682	-0.0000000	0.0983491	-0.0927835	-0.0000003
H	1.6863229	3.4597225	-0.0000031	-0.0419796	-0.2046831	0.0000008
H	-0.4723699	4.6942528	0.0000000	-0.0000285	-0.2253564	0.0000025
H	-2.6312326	3.4600347	-0.0000040	0.0419527	-0.2046667	-0.0000051

ΔE<0, F=2nN LGMF algorithm				Force		
C	-1.6839112	-2.7420902	0.0000026	-0.0185678	-0.2655362	0.0000139
C	-1.7034744	-1.3179408	-0.0000000	-0.0702916	0.0064968	-0.0000129
C	-0.4727418	-0.5817887	-0.0000039	0.0000028	0.0487376	-0.0000116
C	0.7578853	-1.3181171	-0.0000041	0.0702903	0.0064858	-0.0000014
C	0.7381191	-2.7422641	0.0000003	0.0185339	-0.2655445	0.0000030
C	-0.4729465	-3.4437152	0.0000029	-0.0000150	-0.2326557	0.0000125

C	-2.9136780	-0.5438976	0.0000030	0.1433358	-0.1752555	0.0000067
C	-2.9135823	0.8310666	-0.0000035	0.1433692	0.1752283	0.0000040
C	-1.7032680	1.6049386	-0.0000028	-0.0702982	-0.0064941	-0.0000016
C	-0.4726396	0.8686112	-0.0000029	0.0000021	-0.0487383	-0.0000014
C	1.9681987	-0.5442448	-0.0000037	-0.1433710	-0.1752206	-0.0000125
C	1.9682971	0.8307198	0.0000004	-0.1433478	0.1752485	0.0000045
C	0.7580936	1.6047639	0.0000012	0.0702930	-0.0064950	0.0000073
C	0.7385301	3.0289137	0.0000034	0.0185794	0.2655361	0.0000040
C	-0.4724358	3.7305378	-0.0000002	0.0000169	0.2326528	-0.0000086
C	-1.6835010	3.0290855	-0.0000017	-0.0185391	0.2655430	0.0000001
H	-2.6145027	-3.3138854	-0.0000007	-0.0363300	-0.2250736	-0.0000101
H	1.6686287	-3.3141929	-0.0000017	0.0363044	-0.2250905	-0.0000017
H	-0.4730263	-4.5379304	0.0000017	-0.0000243	-0.2321068	-0.0000052
H	-3.8497198	-1.0947293	0.0000101	0.0934721	-0.0836472	0.0000109
H	-3.8495485	1.3820284	-0.0000100	0.0934502	0.0836371	-0.0000146
H	2.9041631	-1.0952081	-0.0000036	-0.0934715	-0.0836382	0.0000030
H	2.9043403	1.3815497	0.0000018	-0.0934501	0.0836521	-0.0000011
H	1.6691215	3.6007097	0.0000101	0.0363432	0.2250809	0.0000076
H	-0.4723587	4.8247530	-0.0000023	0.0000133	0.2321139	-0.0000056
H	-2.6140110	3.6010141	0.0000028	-0.0363001	0.2250834	0.0000109
	**ΔE>0, F=2nN 2nd order PES**				**Force**	
6	-1.6858881	-2.6429522	-0.0000551	0.0447587	0.6351675	-0.0001257
6	-1.7159467	-1.2550766	-0.0000510	0.1813887	0.0289811	-0.0001120
6	-0.4727766	-0.5598896	-0.0000890	0.0000187	-0.1414435	-0.0001022
6	0.7702926	-1.2552569	-0.0000622	-0.1813532	0.0289622	-0.0001735
6	0.7400287	-2.6431260	-0.0000553	-0.0446819	0.6352166	-0.0000760
6	-0.4729790	-3.3253764	-0.0000686	0.0000296	0.5750671	-0.0001430
6	-2.9637131	-0.5326063	0.0000510	-0.3547089	0.4094007	0.0000060
6	-2.9636179	0.8198073	0.0000411	-0.3547663	-0.4093302	-0.0000017
6	-1.7157469	1.5420991	-0.0000549	0.1814059	-0.0289979	-0.0001059
6	-0.4726760	0.8467346	-0.0000854	0.0000568	0.1414440	-0.0000958
6	2.0181608	-0.5329610	0.0000388	0.3547750	0.4093589	0.0000617
6	2.0182567	0.8194519	0.0000458	0.3547149	-0.4094098	0.0000249
6	0.7704922	1.5419254	-0.0000492	-0.1813431	-0.0289216	-0.0001767

6	0.7404242	2.9297988	-0.0000379	-0.0448008	-0.6352072	-0.0000456
6	-0.4724861	3.6122214	-0.0000603	-0.0000648	-0.5750680	-0.0000425
6	-1.6854908	2.9299689	-0.0000543	0.0446874	-0.6352065	-0.0000231
1	-2.6359775	-3.1615104	-0.0000034	0.0562437	0.5893911	0.0001080
1	1.6900377	-3.1618290	0.0000022	-0.0562238	0.5893632	0.0001302
1	-0.4730537	-4.4037631	-0.0000389	0.0000539	0.5710400	0.0000901
1	-3.9031275	-1.0816821	0.0001843	-0.2237827	0.1634062	0.0002177
1	-3.9029563	1.3690165	0.0001507	-0.2238418	-0.1633717	0.0001264
1	2.9575009	-1.0821630	0.0001459	0.2238239	0.1634390	0.0001262
1	2.9576737	1.3685208	0.0001620	0.2237740	-0.1634649	0.0001541
1	1.6905075	3.4483666	0.0000188	-0.0563184	-0.5893747	0.0001176
1	-0.4724098	4.6906073	-0.0000539	-0.0000503	-0.5710630	-0.0000115
1	-2.6355039	3.4486662	-0.0000212	0.0562050	-0.5893782	0.0000724

	**ΔE<0, F=2nN 2nd order PES**				**Force**	
6	-1.6832579	-2.7402586	-0.0000545	-0.0335412	-0.6398263	-0.0000629
6	-1.7027083	-1.3175188	-0.0000243	-0.1574555	0.0090856	-0.0000050
6	-0.4727674	-0.5809174	-0.0000022	0.0000760	0.1278923	-0.0001579
6	0.7570630	-1.3177057	0.0000031	0.1575940	0.0090263	0.0001248
6	0.7373766	-2.7404489	-0.0000424	0.0333551	-0.6398687	-0.0000279
6	-0.4729981	-3.4418917	0.0000260	-0.0001164	-0.5673263	0.0005681
6	-2.9122803	-0.5434438	0.0000595	0.3751321	-0.4234107	0.0002614
6	-2.9121867	0.8306354	0.0000549	0.3751289	0.4233468	0.0002643
6	-1.7025049	1.6045405	-0.0000424	-0.1574733	-0.0090483	-0.0000272
6	-0.4726678	0.8677637	-0.0000218	0.0000494	-0.1279257	-0.0002702
6	1.9667454	-0.5437920	0.0000911	-0.3750591	-0.4233530	0.0004187
6	1.9668409	0.8302925	0.0000436	-0.3749988	0.4234263	0.0002281
6	0.7572678	1.6043761	-0.0000605	0.1575898	-0.0090567	-0.0001040
6	0.7377894	3.0271216	-0.0000827	0.0334707	0.6398660	-0.0000704
6	-0.4724845	3.7287378	0.0000868	-0.0000184	0.5673304	0.0009712
6	-1.6828468	3.0272791	-0.0000552	-0.0334430	0.6398418	0.0000066
1	-2.6122135	-3.3144167	-0.0002421	-0.0547969	-0.5792899	-0.0009345
1	1.6662303	-3.3147731	-0.0002454	0.0545581	-0.5794432	-0.0010386
1	-0.4730926	-4.5357447	0.0001391	-0.0001520	-0.5656632	0.0007833
1	-3.8480703	-1.0938449	0.0001727	0.2528774	-0.1899680	0.0001348

1	-3.8479045	1.3811587	0.0001940	0.2527789	0.1898928	0.0002855
1	2.9024677	-1.0943116	0.0002242	-0.2527587	-0.1898964	0.0001548
1	2.9026389	1.3806847	0.0001576	-0.2527388	0.1899773	0.0002188
1	1.6667284	3.6013076	-0.0003725	0.0547110	0.5794128	-0.0014817
1	-0.4724204	4.8225912	0.0003252	-0.0000511	0.5656612	0.0012742
1	-2.6117194	3.6015715	-0.0003320	-0.0547179	0.5793166	-0.0015144

S_0 minimum Pyrene out plane

6	-1.684758	-2.691987	0.000000
6	-1.709755	-1.286663	0.000000
6	-0.472782	-0.570536	0.000000
6	0.764090	-1.286839	0.000000
6	0.738893	-2.692159	0.000000
6	-0.472982	-3.383879	0.000000
6	-2.938510	-0.537999	0.000000
6	-2.938413	0.825196	0.000000
6	-1.709552	1.573685	0.000000
6	-0.472680	0.857382	0.000000
6	1.992951	-0.538350	0.000000
6	1.993048	0.824845	0.000000
6	0.764294	1.573509	0.000000
6	0.739296	2.978833	0.000000
6	-0.472480	3.670725	0.000000
6	-1.684355	2.979005	0.000000
1	-2.624031	-3.238864	0.000000
1	1.678089	-3.239170	0.000000
1	-0.473059	-4.470041	0.000000
1	-3.877083	-1.086244	0.000000
1	-3.876908	1.373575	0.000000
1	2.931446	-1.086729	0.000000
1	2.931622	1.373090	0.000000
1	1.678569	3.525711	0.000000
1	-0.472402	4.756887	0.000000
1	-2.623550	3.526016	0.000000

ΔE>0, F=2nN LGMF algorithm				Force		
C	-1.6852635	-2.6508530	0.0983714	0.0167620	0.2460508	0.0078794
C	-1.7108912	-1.2630764	0.0252448	0.0999799	-0.0669687	0.0988312
C	-0.4727501	-0.5633935	0.0373378	-0.0000045	-0.1121228	0.0446465
C	0.7652845	-1.2632494	0.0250028	-0.1000114	-0.0669703	0.0986492
C	0.7394798	-2.6510372	0.0979881	-0.0167570	0.2459522	0.0079192
C	-0.4729314	-3.3303879	0.1606276	0.0000607	0.1967822	0.0389761
C	-2.9409934	-0.5318993	-0.1253733	-0.1498308	0.2287372	-0.0215620
C	-2.9408469	0.8190221	-0.1255551	-0.1490527	-0.2289055	-0.0206080
C	-1.7106734	1.5500748	0.0250028	0.0999699	0.0669321	0.0986763
C	-0.4726399	0.8502202	0.0373351	0.0000173	0.1120383	0.0445883
C	1.9954762	-0.5322136	-0.1255156	0.1494987	0.2287704	-0.0210587
C	1.9956008	0.8187090	-0.1253369	0.1497108	-0.2287852	-0.0213897
C	0.7655016	1.5498967	0.0252341	-0.1000318	0.0669867	0.0988509
C	0.7398783	2.9376772	0.0983450	-0.0168363	-0.2459860	0.0079708
C	-0.4724527	3.6172153	0.1606372	-0.0000627	-0.1967935	0.0389487
C	-1.6848709	2.9378653	0.0980247	0.0167237	-0.2460566	0.0078368
H	-2.6280986	-3.1829565	0.0882785	0.0375953	0.1588662	-0.0285627
H	1.6822165	-3.1833053	0.0875488	-0.0376232	0.1588803	-0.0287829
H	-0.4730017	-4.4072039	0.2246820	0.0000592	0.1830662	-0.0085422
H	-3.8602862	-1.0784293	-0.2969711	0.0290899	0.0914679	-0.0941234
H	-3.8600120	1.3656814	-0.2972245	0.0284178	-0.0911808	-0.0946700
H	2.9146579	-1.0788664	-0.2972542	-0.0288421	0.0914037	-0.0943892
H	2.9148939	1.3652529	-0.2969044	-0.0288505	-0.0913659	-0.0941862
H	1.6827151	3.4697793	0.0882188	-0.0376337	-0.1588930	-0.0286221
H	-0.4723761	4.6940345	0.2246580	-0.0000200	-0.1830512	-0.0085447
H	-2.6276088	3.4701374	0.0875963	0.0376717	-0.1588546	-0.0287315

ΔE<0, F=2nN LGMF algorithm				Force		
C	-1.6831901	-2.7149868	-0.0518207	-0.0094854	-0.1507087	-0.0700836
C	-1.7054494	-1.3014058	0.0358416	-0.0468100	0.0141132	0.0832682
C	-0.4727406	-0.5757533	-0.0001164	-0.0000063	0.0394965	-0.0001700
C	0.7598736	-1.3015746	-0.0360408	0.0468366	0.0141696	-0.0834479
C	0.7374086	-2.7151611	0.0514023	0.0094875	-0.1507369	0.0699603
C	-0.4729388	-3.4124322	-0.0002650	0.0000030	-0.1299860	0.0000346

C	-2.9224718	-0.5380000	0.0749161	0.0858841	-0.1057770	0.1678695
C	-2.9224144	0.8251678	-0.0741644	0.0860951	0.1055658	-0.1671994
C	-1.7052578	1.5883922	-0.0359390	-0.0468220	-0.0141726	-0.0830753
C	-0.4726424	0.8625696	-0.0000548	0.0000213	-0.0394972	-0.0001626
C	1.9770221	-0.5383426	-0.0744849	-0.0858559	-0.1058847	-0.1680110
C	1.9770990	0.8247989	0.0748132	-0.0857338	0.1059647	0.1679480
C	0.7600706	1.5882130	0.0359824	0.0468370	-0.0141358	0.0832017
C	0.7378270	3.0018023	-0.0515128	0.0095344	0.1506916	-0.0698649
C	-0.4724395	3.6992518	-0.0003061	-0.0000022	0.1299489	0.0000168
C	-1.6828009	3.0019952	0.0511736	-0.0094797	0.1506848	0.0697030
H	-2.6103914	-3.2689002	-0.1964561	0.0049936	-0.1067681	-0.1945532
H	1.6645223	-3.2692214	0.1960487	-0.0050829	-0.1067593	0.1944772
H	-0.4730126	-4.5030777	-0.0002901	0.0000049	-0.1289594	0.0000874
H	-3.8516280	-1.0773756	0.2407410	0.0839765	-0.0174402	0.3036907
H	-3.8516001	1.3647060	-0.2392221	0.0834282	0.0177249	-0.3035260
H	2.9061765	-1.0778582	-0.2398676	-0.0837485	-0.0175233	-0.3036745
H	2.9062756	1.3641564	0.2406260	-0.0840281	0.0173559	0.3038373
H	1.6650704	3.5557319	-0.1958308	-0.0048647	0.1068287	-0.1942503
H	-0.4723675	4.7898954	-0.0004857	-0.0000286	0.1289265	0.0000096
H	-2.6099683	3.5560959	0.1953120	0.0048461	0.1068781	0.1939146

	**ΔE>0, F=2nN 2nd order PES**				**Force**	
6	-1.212025	-2.835404	0.091653	-0.000379122	0.000311857	-0.000633292
6	-1.237022	-1.430083	0.055245	-0.003963226	0.000650187	-0.002238919
6	-0.000051	-0.713958	0.095093	0.000003781	-0.000167104	-0.002152154
6	1.236818	-1.430259	0.054998	0.003959560	0.000644255	-0.002240368
6	1.211622	-2.835576	0.091220	0.000378021	0.000301078	-0.000628134
6	-0.000251	-3.527295	0.136199	0.000008817	0.000414932	-0.000879510
6	-2.465774	-0.681420	-0.108967	-0.002930597	-0.004642140	-0.000388179
6	-2.465677	0.681771	-0.109162	-0.002939488	0.004649752	-0.000392159
6	-1.236818	1.430259	0.055014	-0.003960908	-0.000644907	-0.002238459
6	0.000051	0.713958	0.095094	-0.000004342	0.000167293	-0.002150907
6	2.465677	-0.681771	-0.109158	0.002938745	-0.004648806	-0.000390911
6	2.465774	0.681420	-0.108926	0.002928663	0.004639093	-0.000388361
6	1.237022	1.430083	0.055256	0.003962015	-0.000650749	-0.002239936

6	1.212025	2.835404	0.091619	0.000379044	-0.000310560	-0.000631722
6	0.000251	3.527295	0.136174	-0.000006922	-0.000414935	-0.000878659
6	-1.211622	2.835576	0.091263	-0.000377961	-0.000303075	-0.000629280
1	-2.151296	-3.382280	0.054484	0.000424287	0.000115978	0.000781189
1	2.150815	-3.382586	0.053635	-0.000432994	0.000119422	0.000786530
1	-0.000328	-4.613455	0.164256	0.000000401	0.000275905	0.000018135
1	-3.404345	-1.229665	-0.289146	0.008597230	0.003566222	0.003977895
1	-3.404170	1.230150	-0.289590	0.008616441	-0.003581413	0.003988946
1	3.404170	-1.230150	-0.289577	-0.008615397	0.003581404	0.003988825
1	3.404345	1.229665	-0.289046	-0.008592315	-0.003563603	0.003975039
1	2.151296	3.382281	0.054426	-0.000424738	-0.000116263	0.000780813
1	0.000328	4.613455	0.164183	-0.000000383	-0.000275059	0.000020008
1	-2.150815	3.382586	0.053761	0.000431387	-0.000118763	0.000783570
	**ΔE<0, F=2nN 2nd order PES**				**Force**	
6	-1.212025	-2.835404	0.033484	-0.002780753	-0.001137648	-0.000778983
6	-1.237022	-1.430083	-0.035774	0.000012406	-0.000224248	0.001671619
6	-0.000051	-0.713958	0.000146	-0.000003517	-0.000322865	-0.000005218
6	1.236818	-1.430259	0.035989	-0.000016357	-0.000220569	-0.001676588
6	1.211622	-2.835576	-0.033213	0.002785745	-0.001144030	0.000775819
6	-0.000251	-3.527295	0.000160	0.000001693	0.000364967	-0.000000705
6	-2.465774	-0.681420	-0.065992	-0.004269159	0.001927690	0.002922471
6	-2.465677	0.681771	0.065600	-0.004249027	-0.001947590	-0.002924821
6	-1.236818	1.430259	0.035908	0.000018653	0.000217093	-0.001670357
6	0.000051	0.713958	0.000094	0.000003874	0.000322661	-0.000003229
6	2.465677	-0.681771	0.065769	0.004259065	0.001951146	-0.002928516
6	2.465774	0.681420	-0.065968	0.004272226	-0.001937758	0.002924207
6	1.237022	1.430083	-0.035863	-0.000015463	0.000219240	0.001669075
6	1.212025	2.835404	0.033317	0.002777859	0.001136788	-0.000775938
6	0.000251	3.527295	0.000167	-0.000001899	-0.000363718	0.000000934
6	-1.211622	2.835576	-0.033121	-0.002773304	0.001138344	0.000777044
1	-2.151296	-3.382280	0.145960	0.002949123	0.001894055	-0.004211217
1	2.150815	-3.382586	-0.145810	-0.002955212	0.001898060	0.004214342
1	-0.000328	-4.613455	0.000130	-0.000000153	-0.000021126	0.000001665
1	-3.404345	-1.229665	-0.205430	0.004196244	0.002825641	0.006837885

1	-3.404170	1.230150	0.204483	0.004160281	-0.002804585	-0.006823284
1	3.404170	-1.230150	0.204853	-0.004172497	0.002812739	-0.006831022
1	3.404345	1.229665	-0.205419	-0.004196184	-0.002826491	0.006840669
1	2.151296	3.382281	0.145683	-0.002942972	-0.001890735	-0.004208222
1	0.000328	4.613455	0.000260	-0.000000185	0.000021002	-0.000000239
1	-2.150815	3.382586	-0.145413	0.002939512	-0.001888065	0.004202608

References:

[S1] Fletcher, R. Practical Methods of Optimization; John Wiley & Sons: New York, 1987.
